# Development and validation of the CAPPAC scale to measure multidimensional play in children and adolescents

**DOI:** 10.1038/s41598-026-43894-x

**Published:** 2026-03-19

**Authors:** Giovanna Pedroni, Matilde Melotto, Elena Pedroni, Sophie Mayen, Anne-Linda Camerini

**Affiliations:** 1 Institute of Public Health, Faculty of Biomedical Sciences, Università della Svizzera italiana, Via Giuseppe Buffi 13, 6900 Lugano, Switzerland; 2 Université Paris 1 Panthéon-Sorbonne, Paris, France

**Keywords:** Play, Digital, Hybrid, Children, Adolescents, Scale development, Confirmatory factor analysis, Parents, Neuroscience, Psychology, Psychology

## Abstract

Play is a core component of human behaviour that supports cognitive, social, and emotional development; yet its assessment remains fragmented across contexts and methodologies. The present study aimed to develop and validate a multidimensional measure of individual play perceptions in children and adolescents. In two independent studies conducted in Switzerland with parents of children aged 4 to 11 and adolescents aged 12 to 15, we performed exploratory (Study 1: *N* = 305) and confirmatory (Study 2: *N* = 263) factor analyses to develop and validate a multidimensional measure of individual play perceptions as part of the Context, Activity, and Perceptions of Play in Adolescents and Children (CAPPAC) scale. Study 1 identified seven underlying dimensions that were confirmed in Study 2 and eventually labelled as: positive immersion, creative engagement, adversarial play, cooperative play, cognitive gain, physical engagement, and autonomous play. The dimensions demonstrated satisfactory psychometric properties, including internal consistency and construct validity. The final CAPPAC scale assesses three domains of play: (1) the context in which play occurs (social, spatial, temporal); (2) the play modality of the activity (analogue, digital, hybrid); and (3) individual play perceptions. By linking contextual, activity-based, and perceptual aspects of play, the CAPPAC scale provides researchers, educators, clinicians, and practitioners with a conceptually grounded and adaptable tool to examine how play, as a multidimensional and situated experience, contributes to developmental processes.

**Supplementary Information:**

The online version contains supplementary material available at 10.1038/s41598-026-43894-x.

## Introduction

Play is a fundamental component of human development, rooted in both biological and cultural evolution, and changes across the lifespan. It functions as an adaptive system that strengthens cognitive flexibility and learning through exploration and problem-solving^1^, supports social bonding and cooperative skill development^2^, and contributes to stress regulation via neurobiological reward pathways^3,4^. Evolutionary perspectives further describe childhood as an extended exploratory period in which play serves as a primary mechanism for acquiring skills, fostering creativity, and developing adaptive strategies for navigating complex environments^5,6^.

Social and technological shifts have expanded the repertoire of play activities available to children and adolescents: their play ranges from traditional board games^7^ and outdoor activities to video games^8,9^ and augmented-reality environments that merge virtual and physical settings^10^. These practices can be broadly grouped into analogue, digital, and hybrid play modality. Yet, play presents an intrinsic multidimensionality, shaped by intrapersonal, interpersonal, and spatiotemporal factors^11^. Thus, play remains closely tied to developmental processes, and its meaning and expression simultaneously depend on the shifting contexts in which children and adolescents participate^12^.

The spontaneous nature of play complicates its systematic study, and this challenge has intensified in the digital era. Analogue play encompasses solitary, cooperative, and competitive formats, whereas digital platforms have expanded these through remote multiplayer interaction, asynchronous engagement, and participation in online communities. Play activities that were once tied to physical environments such as homes and schools, can now be experienced in digital and hybrid spheres that encompass screens, apps, and networked spaces. Furthermore, play has become more fluid, unfolding across breaks, transitions, and daily routines rather than remaining confined to dedicated free time^13^. Digital transformations have reshaped how play is initiated, organized, and experienced, broadening the range of activities^12,14^ and contributing to shorter, on-demand patterns shaped by digital platforms.

While considering the intrinsic multidimensionality of play in context is central to its study, capturing this is far from scientifically straightforward, particularly in controlled settings or with standardized instruments (for a systematic review on pre-school children, see^15^). Existing tools tend to measure only isolated components of play, thereby missing how activity, context, and individual perception jointly influence human play experiences. This gap limits our capacity to determine which specific aspects of play promote particular developmental outcomes^16^.

Understanding how children and adolescents play in contemporary settings—including how they construct and experience play and how they distinguish it from related activities such as media use or social interaction—calls for a validated instrument that can assess the multidimensional character of play across activities, contexts, and subjective psychological perceptions. The present study develops and validates the Context, Activity, and Perceptions of Play in Adolescents and Children (CAPPAC) scale. We aim to provide researchers with a tool that captures complex play experiences, enabling evidence-based insights that can support parents, educators, and practitioners in fostering healthy play that nurtures the development of children and adolescents—today and in future digitally immersed surroundings.

### The concept of play

The concept of play remains elusive and difficult to operationalize, posing a challenge for researchers in developmental and behavioural sciences and any discipline that examines behaviour across naturalistic and structured environments. Despite numerous attempts to specify the defining features of play, empirical research still lacks a unified and comprehensive conceptualization^17^, notwithstanding its recognized importance in human development and emerging evidence that playfulness can act as a psychological resource in demanding or adverse conditions^18^.

Contemporary theories increasingly conceptualize play as a multidimensional and situated experience rather than a set of discrete, observable behaviours. Developmental perspectives, such as Vygotsky’s theory of symbolic play, emphasize the interplay of cognitive, emotional, and social functions within cultural and interpersonal contexts. Building on this foundation, recent multidimensional frameworks^17,19^ further describe play as encompassing emotional, cognitive, social, and physical dimensions. Psychological theories that were not originally developed to explain play, including Flow Theory^20^ and Self-Determination Theory (SDT)^21^, provide additional insight into its experiential and motivational dynamics as they highlight how optimal engagement, intrinsic motivation, and perceived autonomy shape the subjective quality of play. According to SDT, individuals seek to satisfy three basic psychological needs: autonomy (volitional action), competence (feeling effective and capable), and relatedness (feeling connected to others). Play is theoretically well suited to support these needs, thereby linking subjective play experiences to well-being and healthy development (e.g.,^22^).

Although these theories address the play concept only indirectly, they valuably contribute to understanding its underlying processes. Advancing the scientific study of play, however, requires moving beyond its functional or motivational correlates toward a clearer conceptual account of play as an experiential phenomenon. Contemporary models drawn from neuroscience, cognitive science, and philosophy of mind^23,24^ describe human experience as a dynamic, context-sensitive system influenced by the integration of perception, emotion, and bodily states. From this view, play is not simply a set of observable behaviours but a situated experience that unfolds across multiple layers. This experiential dynamic becomes even more complex in digital environments, where technological features add new conditions under which play emerges and evolves.

From a neuroscientific perspective, play can be understood as a dynamic system in which perception, memory, emotion, and decision-making are tightly interconnected^23,25,26^. While these neuroscientific models primarily describe the organization of neural processes rather than play itself, they highlight the strong context sensitivity that characterizes experiential phenomena. This systemic view challenges traditional modular or linear models and instead paves the way for conceptualizing play as context-sensitive and embodied; shaped by the continuous integration of perception, action, and environment^26–28^. In this sense, play can be seen as a phenomenon that exhibits recognisable patterns, while remaining fluid and sensitive to contextual cues. This complex entity displays family resemblance, contextual variability, and a prototype-based organization, properties that can be suitably captured by a dynamic concept theory^26,28,29^.

Recognizing these complexities highlights the need for measures capable of capturing play in a systematic and psychometrically robust manner. Yet most available measures^30–32^ still reduce play to isolated behaviours and overlook its phenomenological complexity.

To address this shortcoming, we draw upon two complementary levels of theoretical analysis: At the conceptual level, play is not a rigid category but a fluid construct, represented by a flow-like state of positive immersion. At the experiential level, play involves different psychological dynamics, such as those described in developmental and motivational theories^20,21^.

### Measuring play: existing approaches and limitations

Existing play measures largely fall into two methodological traditions: The first primarily relies on direct behavioural observation and on parent or teacher reports, focusing on social, developmental, or motor elements of play. These approaches are primarily applied to analogue play. Measures such as the Revised Knox Preschool Play Scale^33^ and the Outdoor Free Play Scale^30^ offer methodologically structured and reliable assessments. However, they do not capture digital play and are typically restricted to younger age groups, particularly pre-schoolers.

The second tradition emerged alongside the expansion of videogaming and relies mainly on self-report measures that quantify variables such as gaming frequency^34,35^, duration^36^ or motivation to play^37^. Research in this area has often shifted towards categorizing experiences as positive or negative^32,38^ or toward identifying clinical or pathological videogaming patterns^39,40^. This narrow lens overlooks play as a complex, multidimensional activity that can encompass both beneficial and challenging human experiences.

Further, the distinction between analogue and digital approaches misses hybrid forms of play that combine analogue (i.e., physical) and digital elements, such as Internet-connected toys, playful interactions with voice assistants like *Siri* or *Alexa*, or play in virtual reality environments. It becomes apparent that these activities remain difficult to assess with existing measures.

Another limitation concerns the assessment strategies themselves. Current tools fall into two categories: observational and other-report measures (for example, parent or teacher reports) and self-reported measures. Observational and other-report measures^30,33,41^ are essential for studying younger children, who have limited metacognitive capacity and cannot yet provide detailed accounts of their own experiences. While these approaches offer valuable behavioural data, they capture only part of the play phenomena as they provide limited aspects of the perceptive experiences of play, such as emotional engagement or intrinsic motivation.

Together, these complexities highlight the conceptual and methodological shortcomings in existing assessment tools. Scientists still lack a measure that reflects how contemporary play unfolds across settings, technologies, and psychological states*.* From this perspective, evaluating play requires understanding not only the modalities and contexts, but also how play is experienced by the individual. Experiential dimensions such as immersion, autonomy, creativity, cooperation, and embodied or competitive dynamics recur across major theoretical accounts of play, including^21,42^; all of which are theoretically central to play but are rarely systematically operationalized in existing measures.

### The present study

In the present study, we introduce the Context, Activity, and Perceptions of Play in Adolescents and Children (CAPPAC) scale. With this scale, we provide a novel tool designed to capture play as a situated and subjective experience.

The CAPPAC scale integrates three conceptual domains: (1) the context of the play activity, including social, spatial, and temporal dimensions; (2) the modality of the play activity, i.e., analogue, digital, and hybrid; and (3) the subjective perceptions associated with the play activity. Specifically, this study aims to develop and psychometrically validate the CAPPAC scale applicable across childhood and adolescence, which allows developmental comparisons within a *single* conceptual structure. To do so, we conducted exploratory and confirmatory factor analyses in two independent samples.

Because younger children have a limited metacognitive capacity, we offer two versions of the scale, a parent-report for children and a self-report for adolescents^43^. To our knowledge, the CAPPAC scale is the first measure to combine context, activity modality, and perceptions within a single, age-inclusive framework. Its design responds to the need for a comprehensive measure that reflects the full scope of contemporary play by drawing on dynamic concept theory, which views constructs as context-sensitive rather than fixed definitions. It aligns with developmental, neurological, and psychological theories, highlighting the interplay of cognition, emotion, embodiment, and context^23,24,26^.

## Method

This section details the steps taken in the development and validation of the domain capturing the subjective perceptions of play captured by the CAPPAC scale. The process followed four stages: (1) item generation informed by ecological observations and theoretical frameworks; (2) identification of the factor structure through exploratory factor analysis (EFA, Study 1); (3) validation of the factor structure using confirmatory factor analysis (CFA, Study 2); and (4) assessment of the scale’s internal consistency and construct validity (Study 2). Study 1 and 2 relied on two independent samples and followed largely the same recruitment and data collection procedures. This work forms part of a broader mixed-methods project into the role of play in children’s and adolescents’ socio-emotional, cognitive, and physical development. The scale development study was preregistered on OSF (10.17605/OSF.IO/D9JZH). All procedures followed established scale development standards^44–46^.

### Item generation

The item generation process comprised four steps. First, we conducted a narrative review of the extant literature on play across developmental psychology, education, and public health studies (1) to map existing observational, other-report, and self-report instruments of play; (2) to identify perceptual, emotional, and cognitive constructs previously linked to play perceptions; and (3) to examine the theoretical frameworks that have guided the operationalization of play in empirical research. Searches were conducted in PsycINFO, PubMed, and Scopus using combinations of terms related to play (e.g., play, game), measurement (e.g., measurement, scale, questionnaire, assessment), and the population of interest (e.g., child, teenager, adolescent). Truncation and wildcards were used to broaden the search. We focused on peer-reviewed publications in English that described measurement approaches to play or play-related experiences. The narrative review was not designed as a formal systematic review but as a theory-informed mapping exercise supporting item generation. Overall, the review confirmed the absence of a unified, multidimensional tool capable of capturing children’s and adolescents’ subjective perceptions of play across diverse settings and formats^31,47,48^. At the same time, it generated an initial list of candidate dimensions of play perceptions and informed the wording and structure of items for operationalization. These theoretically derived insights were complemented by qualitative focus groups, which provided experiential depth and age-appropriate linguistic formulations grounded in children’s and adolescents’ contemporary play contexts.

Second, we drew from a series of qualitative focus groups with children and adolescents aged 4 to 15 years, conducted as part of a broader project on OSF (10.17605/OSF.IO/H6WZU). These discussions explored how children and adolescents define play, along with their preferences, motivations, and barriers and enablers to play across settings. We identified 13 core dimensions of play perceptions by integrating insights from the literature review and the focus groups. These dimensions were: fun, attention, emotional activation, cognition, freedom, creativity, imagination, movement, competition, cooperation, pro-sociality, aggression, and spontaneity.

Third, for each dimension, we collaboratively generated five items, also consulting additional language reference materials to ensure semantic breadth and clarity. This step resulted in an initial pool of 65 items. We prioritized clarity, age-appropriateness, and variation to minimize potential response biases such as ceiling or floor effects.

Fourth, four members of the research team independently reviewed the 65-item pool to assess conceptual relevance, clarity of wording, and redundancy, resulting in minor refinements and improved consistency across the dimensions. We then invited four external experts in education, philosophy, and psychology, who had not participated in earlier stages, to evaluate the content validity of the revised item set. We briefed experts on the theoretical definitions of the constructs and asked them to evaluate the alignment and representativeness of each item. The definitions of the constructs consisted of brief, theory-based descriptions derived from the integration of the literature review and focus groups. They articulated the central meaning of each construct within the play context and served as a guide for the experts in assessing item-construct alignment, representativeness, and redundancy. The expert feedback led to further minor wording adjustments until we reached a consensus. We applied a five-point Likert scale (0 = strongly disagree; 4 = strongly agree) to rate each item in the final pool of items (Supplement, Table S1), which served as the basis for subsequent psychometric validation.

### Participants and procedure

Study 1 and 2 were performed in accordance with the relevant guidelines and regulations from the ethics committee of Università della Svizzera italiana, who approved the studies (decision number CE_2025_01). Both studies included data collection through online questionnaires from parents of children aged 4 to 11 and adolescents aged 12 to 15. Before data collection, all families received detailed study information. Parents responding for their children provided informed consent directly at the beginning of the questionnaire. Similarly, adolescents provided informed consent directly at the beginning of the online questionnaire. Since Study 1 and 2 fall under the category of studies with healthy human subjects, with minimal risks (e.g., fatigue during survey completion), and no personally identifiable information was collected, active parental consent was not obtained.

The questionnaires (a parent-report version for children and a self-report version for adolescents) were identical apart from minor wording adjustments (third-person vs. first-person phrasing). We recruited participants in collaboration with the education administration of Canton Ticino, Italian-speaking Switzerland. We randomly selected schools and classes to ensure balanced demographic representation. We collected data between March and April 2025 using an online questionnaire administered through Qualtrics. Participating pre- and elementary schools distributed the survey link to parents by email for completion at home. Parents could freely decide who among the two would respond to the questionnaire. Adolescents in middle school completed the questionnaire on PCs or laptops provided by the schools during a lesson. Data collected was supported by members of the research team who assisted with setup and technical questions. Across the two studies, a total of 568 participants were included.

Study 1 comprised 305 participants, including 149 (48.9%) parents reporting on children aged 4–11 years and 156 (51.1%) adolescents aged 12–15 years. Across both samples, 155 (50.8) were male, 147 (48.2%) were female, and 3 (1%) did not disclose the gender. The majority of the children and adolescents had Swiss nationality (n = 217; 71.2%) and they had just or more than enough to live on (cumulative n = 143; 79.7%). Sixty-eight (22.3%) children and adolescents lived in a single-parent household, and 85 (27.98%) were the only child.

Study 2 comprised a newly recruited sample of 263 participants, including 143 (54.4%) parents reporting on children aged 4–11 years and 120 (45.6%) adolescents aged 12–15 years. Across both samples, 139 (52.9) were male, 119 (45.2%) were female, and 5 (1.9%) did not disclose the gender. Once again, the majority had Swiss nationality (n = 188; 71.5%) and had just or more than enough to live on (cumulative n = 222; 84.4%). Forty-eight (18.3%) children and adolescents lived in a single-parent household, and 78 (29.7%) were the only child.

Detailed descriptive information on demographic characteristics is reported in Table 1 (Study 1) and Table 2 (Study 2).

**Table 1:** Demographic characteristics in Study 1 (EFA).

Demographic characteristics		Total (n = 305, 100%)		Children (n = 149, 48.9%)		Adolescents (n = 156, 51.1%)	
		*n*	%	*n*	%	*n*	%
Study 1: EFA (N = 305)							
Gender	Male	155	50.8	75	50.3	80	51.3
	Female	147	48.2	71	47.7	76	48.7
	Prefer not to say	3	1.0	3	2.0	0	0.0
Nationality	Swiss	217	71.2	122	81.9	95	60.9
	Non-Swiss	40	13.1	15	10.1	25	15.9
	Dual citizenship	48	15.7	12	8.1	36	23.1
Socioeconomic status	More than enough to live on	121	39.7	66	44.3	55	35.3
	Just enough to live on	122	40.0	61	40.9	61	39.1
	Not enough to live on	12	3.9	9	6.0	3	1.9
	Missing	50	16.4	13	8.7	37	23.7
Household composition	Both parents	237	77.7	118	79.2	119	76.3
	Single parent	68	22.3	31	20.8	37	23.7
	Siblings	227	74.4	104	69.8	123	78.8
	Only child	78	25.6	45	30.2	33	21.2
	Other (e.g., grandparents)	11	3.6	5	3.4	6	3.8

Children: individuals aged 4–11 years (parent-report); adolescents: individuals aged 12–15 years (self-report); Multiple answers for household composition (child/adolescent lives with…).

**Table 2:** Demographic characteristics in Study 2 (CFA).

Demographic characteristics		Total (n = 263, 100%)		Children (n = 143, 54.4%)		Adolescents (n = 120, 45.6%)	
		*n*	%	*n*	%	*n*	%
Study 2: CFA (*N* = 263)							
Gender	Male	139	52.9	64	44.8	55	45.8
	Female	119	45.2	79	55.2	60	50.0
	Prefer not to say	5	1.9	0	0.0	5	4.2
Nationality	Swiss	188	71.5	113	79.0	75	62.5
	Non-Swiss	49	18.6	23	16.1	26	21.7
	Dual citizenship	26	9.9	7	4.9	19	15.8
Socioeconomic status	More than enough to live on	123	46.8	76	53.1	47	39.2
	Just enough to live on	99	37.6	54	37.8	45	37.5
	Not enough to live on	37	14.1	9	6.3	28	23.3
	Missing	4	1.5	4	2.8	0	0.0
Household composition	Both parents	215	81.7	126	88.1	89	74.2
	Single parent	48	18.3	17	11.9	31	25.8
	Siblings	185	70.3	99	69.2	86	71.7
	Only child	78	29.7	44	30.8	34	28.3
	Other (e.g., grandparents)	20	12.3	2	1.4	18	15.0

Children: individuals aged 4–11 years (parent-report); adolescents: individuals aged 12–15 years (self-report); Multiple answers for household composition (child/adolescent lives with…); In Study 1, SES showed > 20% missing data. Therefore, in Study 2, SES was set as a mandatory survey question, resulting in reduced missingness.

The questionnaire consisted of three sections: (A) sociodemographic information, (B) social, spatial, and temporal context of play and play modality, and (C) the complete set of 65 play perception items generated in the earlier stages. Because play perceptions are tied to specific play activities, participants were first asked to name their three favourite play activities. The system then randomly selected one of these activities, and all subsequent questions, including the perceptual items, referred to this selected activity.

## Results

### Study 1: exploratory factor analysis

Exploratory factor analyses were conducted on the whole sample including both children and adolescents as the primary goal was to identify a universal latent structure of play perceptions rather than to examine age-specific differences in play contexts or preference for a play modality. The retained factors thus include cross-cutting experiential play dimensions that may occur across different contexts and developmental stages. We performed all data analyses in R Studio (version 4.3.2 (2023–10-31)) using the *psych*^49^ and *lavaan*^50^ packages. Prior to estimating the factor structure, we screened items for missing data, inconsistent responses, and outliers. We defined ceiling and floor effects as more than 20% of responses at the minimum or maximum scale point. Two items (Q6_2 and Q9_1, see Supplement, Table S1) met this criterion, were flagged for review, and excluded from the Study 1 item pool.

For each of the 13 theoretical dimensions derived from the literature review and focus groups, we calculated item-total correlations to identify the three most representative items. We then assessed the internal consistency of these item sets using Cronbach’s alpha. The Shapiro–Wilk test revealed significant deviations from normality, which informed our decision to use robust estimation methods for subsequent analyses.

To examine the underlying factor structure, we estimated two models: (1) a reduced item set comprising the three strongest indicators per theoretical dimension (39 items) selected via item-total correlations, and (2) the full 65-item pool. Factor retention was guided by parallel analysis, eigenvalues > 1 (Kaiser’s criterion), and visual inspection of the scree plot. We extracted factors using minimum residual estimation (minres) with oblimin rotation, which is suitable for non-normal distributions and expected inter-factor correlations. Item-retention followed predefined criteria: (1) primary factor loadings of ≥ .40, (2) no substantial cross-loadings, (3) a minimum of four items per factor, (4) preference for items with higher loadings when multiple candidates were available, and (5) overall parsimony of the item set.

Table 3 summarizes the descriptive statistics of play context and modality in Study 1. Concerning the social context, participants most frequently reported playing with parents (27.6%) or friends (24.2%), followed by siblings (20.6%). Solitary play accounted for 14.2% and other social partners for 13.4%. Play occurred mainly at home (69.8%), with smaller proportions reporting outdoors (8.2%), at someone else’s home (7.5%), or in other locations (14.4%). Most play activities took place after school (34.8%) or in the evening (29.2%), followed by weekends (19.3%). Morning play and other times of day were less common (5.2% and 13.4%). In terms of play modality, about half of the reported activities were analogue (69.8%), digital play represented 24.9% of cases, and hybrid play accounted for 5.2%.

**Table 3:** Descriptive statistics of play context (social, spatial, and temporal) and play modality (analogue, digital, hybrid) in Study 1.

Context and modality		*n*	%
Study 1 (EFA): *N* = 305			
Social context	Friends	114	24.2
	Parents	130	27.6
	Siblings	97	20.6
	Alone	67	14.2
	Other	63	13.4
Spatial context	At my home	213	69.8
	Outdoor	25	8.2
	At someone else’s home	23	7.5
	Other	44	14.4
Temporal context	After school	100	34.8
	In the evening	89	29.2
	At weekend	59	19.3
	In the morning (wk)	16	5.2
	Other	41	13.4
Play modality	Analogue	213	69.8
	Digital	76	24.9
	Hybrid	16	5.2

For social context, *n* = 471, as multiple responses were possible; wk = weekend.

Based on the reduced set of 39 play perception items, the Kaiser–Meyer–Olkin (KMO) index indicated strong sampling adequacy (KMO = .87). Bartlett’s test of sphericity was significant, χ^2^(741) = 5412.88, *p* < .001, confirming that the data were suitable for factor analysis. Parallel analysis and scree plot converged on a seven-factor solution with 28 items, cumulatively explaining 43.1% of the total variance. The proportion of variance accounted for by each factor ranged from 3.8 to 8.3% (Table 4). Standardized factor loadings of the 39 items are provided in the Supplement, Table S2.

**Table 4:** List of 28 items and seven latent factors emerged from the EFA. Proportion of variance attributed to individual factors. Correlation among factors.

#Item	Item description	Positive stimulation	Creativity and imagination	Competition and disruption	Cooperation and pro-sociality	Cognitive stimulation	Movement	Autonomy and spontaneity
1	I enjoyed the moment	.635						
2	I was enthusiastic	.591						
3	I was excited	.484						
4	I was stimulated	.428						
5	I created something		.771					
6	I used artistic skills		.631					
7	I used my imagination		.470					
8	I invented something		.746					
9	I took care of someone/something		.471					
10	I was in competition			.635				
11	I felt challenged			.699				
12	I felt like subduing others			.802				
13	I felt like destroying others			.821				
14	I cooperated with someone				.747			
15	I acted together with someone				.778			
16	I made decisions with someone				.779			
17	I helped someone				.595			
18	I did good for others				.432			
19	I learned something					.736		
20	I acquired knowledge					.741		
21	I understood something					.412		
22	I made a movement						.793	
23	I moved						.805	
24	I changed position						.588	
25	I could choose what to do							.669
26	I could do whatever I wanted							.603
27	I was free							.336
28	I was spontaneous							.380
Eigenvalue		2.988	3.003	3.229	2.566	1.733	1.818	1.479
% of variance		.077	.077	.083	.066	.044	.047	.038
Cumulative %		.077	.154	.236	.302	.347	.393	.431
Correlation among factors	Positive immersion	1.00						
	Creativity and imagination	.178**	1.00					
	Competition and disruption	–.059	–.193**	1.00				
	Cooperation and pro-sociality	.243**	.131**	.161**	1.00			
	Cognitive stimulation	.465**	.264**	–.013	.279**	1.00		
	Movement	.201**	.288**	–.013	.301**	.305**	1.00	
	Autonomy and spontaneity	.330**	.361**	–.142**	.016	.263**	.168**	1.00
Descriptive statistics for each factor	M (SD)	4.10 (0.58)	3.24 (0.58)	2.57 (1.05)	3.17 (1.00)	3.55 (0.94)	3.58 (1.06)	3.96 (0.76)
	α	.861	.699	.867	.823	.780	.802	.703

** Significant correlation *p* < .05.

Applying the predefined retention criteria (primary loadings ≥ .40, no substantial cross-loadings, theoretical coherence, minimum of four items per factor, preference for higher loadings, and parsimony) yielded a structure of 28 items loading on seven latent dimensions: ‘positive stimulation’, ‘creativity and imagination’, ‘competition and disruption’, ‘cooperation and pro-sociality’, ‘cognitive stimulation’, ‘movement’, and ‘autonomy and spontaneity’. Internal consistency was acceptable across all dimensions (Cronbach’s α > .70). Replication of the EFA on the full set of 65 items (Supplement, Table S3) produced a more diffuse structure, with thirty items showing low or cross-loadings and limited correspondence with the theoretical model. Given the clearer structure and stronger theoretical alignment of the reduced solution, we proceeded with the 39-item pool that resulted in the final set of 28 items and seven factors.

### Study 2: confirmatory factor analysis

The online questionnaire of Study 2 included 28 play perception items retained after Study 1 (EFA). We also added 18 newly formulated items, resulting in a 46-item pool (Supplement, Table S4). Although this specific step was not preregistered, it was grounded in two considerations: First, the ongoing review of play measurement literature indicated that several perceptual dimensions, particularly those relevant to analogue play, remained underrepresented in the retained item set. Second, continued analysis of the focus group data, initiated before Study 2 to support the original 65-item pool, revealed additional experiential nuances within these dimensions that warranted more precise operationalization. We developed these items using the same conceptual framework and item-construction criteria as the initial 65-item pool and intended to more fully assess the scope of the experiential dimensions already identified, rather than to introduce new constructs. This strategy is consistent with established guidelines that emphasize theoretical completeness and iterative refinement in scale development^44–46^.

As in Study 1, all analyses were performed in R Studio (version 4.3.2 (2023-10-31)) using functions from the *psych* and *lavaan* packages. Before running the CFA, we screened all play perception items for missing data, inconsistent responding, and outliers. Ceiling and floor effects were again defined as more than 20% of responses at the minimum or maximum scale point. In contrast to Study 1, no items met this criterion, and the full set was retained for analysis.

Next, we verified item variability by ensuring all standard deviations exceeded .50. We assessed intra-factor coherence by flagging items with average inter-item correlations < .30 and checked cross-loadings using correlations > .40 with non-target factors. Sampling adequacy was confirmed with an overall KMO score of .83, and all items surpassed the individual threshold of .60. Based on these criteria, we retained items with clearer alignment to the intended constructs.

To confirm the factorial structure identified in Study 1, we evaluated model fit using multiple indices: the Comparative Fit Index (CFI), Tucker-Lewis Index (TLI), Root Mean Square Error of Approximation (RMSEA), and Standardized Root Mean Square Residual (SRMR). We considered values of CFI ≥ .95, TLI ≥ .90, RMSEA ≤ .06, and SRMR ≤ .08 as indicators of good model fit^51^. We inspected standardized factor loadings and considered items with factor loadings < .40 or limited theoretical relevance for removal. Reliability was assessed with Cronbach’s alpha and corrected item-total correlations, using α ≥ .70 as the criterion for acceptable internal consistency. Content validity had been established during earlier expert review phases, and construct validity was examined through CFA.

Table 5 presents the descriptive statistics of play contexts and modality reported by participants in Study 2 (*N* = 263). Most play activities were carried out with friends (35.7%), followed by parents (28.9%) and siblings (23.4%). Other social partners accounted for 12.0% of responses. Furthermore, play occurred mainly in indoor settings (66.2%), particularly at home (55.5%), with smaller proportions reported at someone else’s home (8.0%) or in public indoor spaces such as gyms or shopping centres (2.7%). Outdoor play represented 33.8% of activities, most often in public spaces such as parks or fields (18.6%), followed by playgrounds (7.6%) and private gardens (4.2%). Just over half of the reported play took place on weekdays (54.4%), predominantly after school (29.7%) and in the evening (16.3%). Weekend play accounted for 45.6%, mostly in the afternoon (28.5%) and evening (11.4%). Finally, most play activities were played in analogue (76.8%) modality, whereas digital play accounted for 21.7%. Hybrid play was rare (1.5%).

**Table 5:** Descriptive statistics of play context (social, spatial, and temporal) and play modality (analogue, digital, hybrid) in Study 2.

Context and modality		*n*	%
Study 2 (CFA): *N* = 263			
Social context	Friends	104	35.7
	Parents	76	28.9
	Siblings	76	23.4
	Other	108	12.0
Spatial context	*Indoor*	174	66.2
	At my home	146	55.5
	At someone else’s home	21	8.0
	Public space	7	2.7
	*Outdoor*	89	33.8
	Public space (od)	49	18.6
	Playground	20	7.6
	Private garden	11	4.2
	Sport centre	9	3.4
Temporal context	*During the weekday*	143	54.4
	In the afternoon	78	29.7
	In the evening	43	16.3
	*Weekend*	120	45.6
	In the afternoon (wk)	75	28.5
	In the evening (wk)	30	11.4
Play modality	Analogue	202	76.8
	Digital	57	21.7
	Hybrid	4	1.5

For social context, *n* = 364, as multiple responses were possible; ‘Other’: e.g., classmate, grandmother; Spatial context *Indoor* ‘public space’: e.g., gym, shopping centre; *Outdoor ‘*public space’: e.g., field, park; od = outdoor; wk = weekend.

Based on psychometric and theoretical criteria, we retained a subset of 23 items from the original 46 items for further analysis. The final CFA model showed satisfactory fit, CFI = .956, TLI = .947, RMSEA = .042 (90% CI [.029, .054]), and SRMR = .055. Standardized factor loadings ranged from .59 to .88, with the lowest loading observed for item #27 within the ‘autonomy and spontaneity’ dimension identified in Study 1. Drawing on loading patterns and conceptual coherence, we confirmed and slightly relabelled seven related play constructs: positive immersion, creative engagement, adversarial play, cooperative play, cognitive gain, physical engagement, and autonomous play (Table 6).

**Table 6:** Results of the confirmatory factor analysis showing factor structure, item descriptions, unstandardized estimates, standard errors, z-values, and standardized loadings (Std.lv and Std.all) for each of the seven latent factors.

Factor	Item description	Estimate	Std.Err	z-value	P( >|z|)	Std.lv	Std.all
Positive immersion	I enjoyed the moment	1				.58	.668
	I was enthusiastic	1.157	.122	9.47	0	.671	.787
	I was stimulated	1.268	.174	7.28	0	.736	.656
	I was totally focused on what I was doing	1.264	.198	6.386	0	.734	.742
Creative engagement	I created something	1				1.089	.748
	I used my artistic skills	.887	.079	11.246	0	.966	.703
	I used my imagination	.947	.079	12.013	0	1.032	.755
	I invented something	1.147	.077	14.841	0	1.25	.881
Adversarial play	I was in competition	1				1.011	.708
	I felt challenged	.985	.121	8.144	0	.996	.702
	I felt the urge to destroy others	.914	.133	6.85	0	.924	.709
Cooperative play	I cooperated with someone	1				.887	.635
	I acted together with someone	1.07	.122	8.779	0	.949	.721
	I made decisions with someone	.981	.114	8.633	0	.87	.663
	I helped someone	.989	.115	8.566	0	.878	.714
Cognitive gain	I learned something	1				.932	.803
	I acquired knowledge	1.007	.094	10.756	0	.938	.814
	I understood something	.924	.079	11.716	0	.861	.773
Physical engagement	I made a movement	1				1.185	.896
	I moved	.809	.351	2.304	.021	.959	.726
Autonomous play	I could choose what to do	1				.859	.817
	I could do what I wanted	1.115	.126	8.847	0	.957	.763
	I was free	.692	.108	6.411	0	.595	.627

We assessed reliability of each latent construct using Cronbach’s alpha and corrected item-total correlations, applying α ≥ 0.70 as the threshold for acceptable internal consistency. Table 7 shows the correlations among the seven dimensions.

**Table 7:** Correlation among the seven factors emerged from the CFA. ** = significant correlation *p* < .05.

Factor	A	B	C	D	E	F	G
Positive immersion (A)	1						
Creative engagement (B)	.420**	1					
Adversarial play (C)	.091	-.303**	1				
Cooperative play (D)	.298**	.135	.164	1			
Cognitive gain (E)	.663**	.551**	-.142	.291**	1		
Physical engagement (F)	.203**	.031	.075	.284**	-.029	1	
Autonomous play (G)	.439**	.399**	-.147	.149	.311**	.089	1
M (SD)	4.07 (0.75)	3.28 (1.17)	2.47 (1.13)	3.38 (1.02)	3.64 (1.00)	3.59 (1.20)	3.98 (0.91)
α	.788	.852	.747	.776	.839	.788	.769

We examined structural comparability across developmental stages by performing multi-group CFAs comparing children (parent-report) and adolescents (self-report). The originally confirmed seven-factor model showed estimation instability in the adolescent subgroup due to the two-item ‘physical engagement’ factor. Hence, invariance testing was conducted on the remaining six factors. The six-factor model showed acceptable fit in both adolescents (CFI = .940, RMSEA = .047, SRMR = .062) and children (CFI = .948, RMSEA = .051, SRMR = .070). A configural invariance model demonstrated satisfactory overall fit (CFI = .944, RMSEA = .049, SRMR = .063), indicating that the same factor structure was justifiable across age groups. Constraining factor loadings to equality (metric invariance) resulted in a small decrease in fit (CFI = .932, RMSEA = 0.053, SRMR = .072), with ΔCFI =  − .012 and ΔRMSEA = .004. Although the decrease in CFI slightly exceeded the conventional − .01 guideline, the change in RMSEA remained minimal^52^. Scalar invariance was not tested as we aimed to evaluate structural comparability.

## Discussion

This paper presents the CAPPAC scale as a multidimensional measure of play that integrates three domains: (1) the context of play, including social, spatial, and temporal dimensions; (2) the play modality, differentiating between analogue, hybrid, and digital play; and (3) the individual perceptions associated with play. We identified and confirmed seven dimensions of play perception: positive immersion, creative engagement, adversarial play, cooperative play, cognitive gain, physical engagement, and autonomous play.

Prior research recognizes play as a dynamic and situated experience that spans a continuum of cognitive, social, emotional, and physical processes^19^. In contemporary settings, play cannot be reduced to fixed activity categories or observed behaviours, since it varies substantially across contexts, activities, and developmental stages^17^. However, existing measures tend to capture only isolated play components, thereby omitting their contextual and experiential complexity. The present research addresses this gap by empirically validating a multidimensional framework of play perceptions. Studies 1 (EFA) and 2 (CFA) support the scale’s construct validity, providing converging evidence that the CAPPAC scale can systematically assess these perceptions across diverse settings and activities.

Several dimensions warrant closer examination. One notable result is the simultaneous emergence of ‘cooperative play’ and ‘adversarial play’. These factors are distinct but not mutually exclusive, which indicates that prosocial and competitive dynamics can co-occur within a single play episode. Ethological research on social play supports this pattern, particularly in studies of rough-and-tumble interactions in mammals, where cooperative and antagonistic elements unfold together, jointly contributing to socio-cognitive development^53^. Rough-and-tumble play, often described as “play of fight and pursuit”, includes behaviours such as wrestling, chasing, and mock fighting. Far from being purely antagonistic, these interactions enable children to experiment with boundaries of both cooperation and conflict, while also providing an outlet for emotional expression and regulation^15^. Likewise, developmental studies show that even structured games can elicit both collaborative and antagonistic behaviours in children^54^. Play should therefore not be conceptualized as intrinsically harmonious or conflictual, but rather as an occasion for exploring and regulating diverse modalities of social interaction.

Second, the dimension ‘positive immersion’, defined by focused attention, emotional stimulation, and enthusiasm, aligns closely with the concept of flow, a state of optimal experience driven by intrinsic motivation and deep engagement^20^. A flow state occurs when individuals perceive an optimal balance (i.e., a “sweet spot”) between challenge and skill, remain fully absorbed in the activity, and engage for its own sake rather than for external outcomes^55^. The positive correlation between ‘positive immersion’ and ‘cognitive gain’ supports this interpretation, indicating that immersive experiences and perceived skill development co-occur during play. Although the items in these dimensions capture central flow markers, specifically the ‘positive immersion’ construct extends beyond flow by also incorporating elements of pure enjoyment and excitement, suggesting that immersion functions as a broader experiential mechanism within children’s and adolescents’ play^56^.

Third, the emergence of ‘creative engagement’ and ‘cognitive gain’ as distinct yet complementary dimensions indicates that play supports both imaginative elaboration and perceived learning. ‘Creative engagement’ reflects symbolic and generative aspects of play, consistent with Vygotsky’s view of its role in developing abstract and representational thinking^42^. In contrast, the dimension ‘cognitive gain’ captures perceived acquisition of knowledge and understanding through exploration. This distinction aligns with current models that describe play as a multidimensional experience integrating affective, cognitive, and creative processes^19^.

Fourth, the emergence of a ‘physical engagement’ dimension highlights the persistent role of bodily activity in play, even among digitally immersed cohorts such as the so-called Gen Z and Gen Alpha. Recent evidence suggests that children continue to spend a significant portion of their playtime outdoors, underscoring the enduring developmental value of unstructured physical play despite the growing influence of digital environments on leisure and interaction^57,58^.

Finally, the ‘perceived autonomy’ dimension, which reflects volition, choice, and self-direction, corresponds directly to the autonomy component of Self-Determination Theory^21^. In this, autonomy is considered a core psychological need that supports intrinsic motivation. Quantitative studies have demonstrated that adolescents report markedly higher autonomy in unstructured or freely chosen activities compared with organized settings^59^. In line with this, our results acknowledge autonomy as a central psychological ingredient of play perceptions. In this sense, self-initiated and freely chosen play likely promotes a sense of ownership and personal agency, enhancing engagement and fostering youths’ meaningful play experiences. Perceived autonomy also contributes to the development of self-regulatory capacities and enables children to navigate challenges, negotiate rules, and make purposeful decisions during play^60^.

It is important to note that, although the identified dimensions of play perception were modelled as common across the age range of the sample, their developmental significance likely varies. Additional invariance analyses indicated substantial structural comparability across children and adolescents for the core dimensions of the scale. At the same time, the ‘physical engagement’ dimension proved more sensitive in age-specific estimation, which may reflect both statistical vulnerability of two-indicator latent variables and developmental differences in how bodily involvement in play is experienced and expressed. Prior research suggests that physical play changes in form, social function, and gender patterning across childhood and adolescence^61–63^. How we operationalized ‘physical engagement’ may warrant further refinement, as any form of movement may be more appropriately assessed using frequency- or intensity-based response formats (e.g., “I moved *a lot*”). Future studies should examine whether alternative wording or scaling improves the stability of this dimension across developmental groups.

Perceived autonomy may likewise shift in meaning across developmental stages, reflecting an emerging sense of self-determination and freedom of choice in younger children, whereas in adolescence it may be more closely linked to identity exploration and independence from adult structures^64^. Overall, the CAPPAC scale provides a shared structural framework across childhood and adolescence, while inviting continued examination of developmental differentiation and measurement refinement, particularly during periods of rapid change such as the transition into early adolescence and puberty.

In conclusion, the seven play perception dimensions provide a theoretically grounded and empirically supported framework for characterizing how children and adolescents experience play. By moving beyond categorical distinctions between analogue, digital, and hybrid play activities, the CAPPAC scale enables a more fine-grained and phenomenologically sensitive assessment as it unfolds across social, temporal, and spatial contexts and developmental stages, in line with contemporary cognitive and developmental science. It reframes play as an open, context-sensitive conceptual structure and allows modelling play in accordance with dynamic theory of concepts^27,28,65^.

### Limitations and implications for future research

Our work has some limitations that warrant consideration. First, the pool of play perception items was administered at a single time point in each study, which prevents an assessment of test–retest reliability. This limits conclusions about the temporal stability of the measurement model of play perceptions, an aspect that will be important for studies aiming to track change over time in longitudinal and intervention-based research. Second, for children aged 4 to 11years we relied on parent reports, a pragmatic choice given developmental constraint in younger children. However, this approach may only partially reflect children’s subjective experience of play. Third, although the samples were diverse in age and background, all data were drawn from Canton Ticino, Italian-speaking Switzerland, which may limit generalizability to other cultural or socio-demographic contexts. The factor structure was replicated in Study 2, and supplementary multi-group analyses indicated structural comparability across age groups. Yet, full measurement invariance across developmental stages, languages, and cultural settings remains to be established. We suggest future research to extend these analyses to test scalar and cross-cultural invariance, and to evaluate test–retest reliability and sensitivity to change in pre–post intervention designs, such as school-based or therapeutic play programs. Longitudinal designs will also be helpful in establishing temporal stability and for evaluating the scale’s responsiveness in intervention contexts. Finally, person-centred analyses, such as latent profile analysis, can further clarify whether distinct engagement patterns emerge across the seven dimensions. These efforts would not only deepen the conceptual understanding of play as a multifaceted, evolutionarily rooted, and contextually shaped human experience but also strengthen the scale’s theoretical and practical relevance across developmental, educational, and clinical research.

## Conclusion

The CAPPAC scale provides a structured approach to measuring play as a situated and multidimensional experience of children and adolescents across social, spatial, and temporal contexts. By integrating individual perceptions of play, activities, and contextual features within a single framework, the scale holds application in developmental science, cognitive and behavioural sciences, education, and child health, including clinical practice. The seven validated play dimensions allow researchers to analyse play as a dynamic construct that varies across settings and age groups, facilitating the assessment of developmentally meaningful, engaging, and psychologically rich play experiences.

Moreover, the CAPPAC scale enables the tracking of individual developmental trajectories over time, thereby fostering the scientific study of how play relates to learning, well-being, health, and development^66,67^ with greater conceptual precision. Its sensitivity to contextual and experiential nuances makes the scale particularly useful in educational settings, where the quality of play is increasingly recognized as a determinant of learning and social development^19^. The CAPPAC scale accounts for the complexity of human play experience while also providing a structured tool for advancing evidence-based research and practice across developmental, educational, and clinical domains.

## Supplementary Information

Supplementary Information.

## Data Availability

The study materials and R codes used in this paper are available in the Open Science Framework (OSF) repository, 10.17605/OSF.IO/D9JZH, along with an online Supplement containing additional tables.
