# Supplementary material for: Development and validation of the CAPPAC scale to measure multidimensional play in children and adolescents
Source: Sci Rep. 2026 Mar 19;16:17651. doi: 10.1038/s41598-026-43894-x (PMC13243616; doi:10.1038/s41598-026-43894-x)
Supplement: Supplementary file 1 — Supplementary Information. [file 41598_2026_43894_MOESM1_ESM.docx]

Supplemental Tables S1—S4

| **Dimension** | **Item description** | **Item** |
| --- | --- | --- |
| Fun | I had fun. | Q1_1 |
|  | I had a good time. | Q1_2 |
|  | I liked it. | Q1_3 |
|  | I enjoyed the moment. | Q1_4 |
|  | I relaxed. | Q1_5 |
| Attention | I was concentrated. | Q2_1 |
|  | I was attentive. | Q2_2 |
|  | I was focused. | Q2_3 |
|  | I was alert. | Q2_4 |
|  | I was clear-headed. | Q2_5 |
| Emotional activation | I was stimulated. | Q3_1 |
|  | I was excited. | Q3_2 |
|  | I was enthusiastic. | Q3_3 |
|  | I was thrilled. | Q3_4 |
|  | I was energetic. | Q3_5 |
| Cognition | I learned something. | Q4_1 |
|  | I thought about something. | Q4_2 |
|  | I reflected on something. | Q4_3 |
|  | I acquired knowledge. | Q4_4 |
|  | I understood something. | Q4_5 |
| Freedom | I was free. | Q5_1 |
|  | I had no constraints. | Q5_2 |
|  | I was autonomous. | Q5_3 |
|  | I could choose what to do. | Q5_4 |
|  | I could do whatever I wanted. | Q5_5 |
| Creativity | I created something. | Q6_1 |
|  | I built something. | Q6_2 |
|  | I used my imagination. | Q6_3 |
|  | I experimented. | Q6_4 |
|  | I used artistic skills. | Q6_5 |
| Imagination | I used my imagination. | Q7_1 |
|  | I had an open mind. | Q7_2 |
|  | I thought freely. | Q7_3 |
|  | I thought outside the box. | Q7_4 |
|  | I invented something. | Q7_5 |
| Movement | I moved. | Q8_1 |
|  | I changed position. | Q8_2 |
|  | I moved my hands. | Q8_3 |
|  | I moved around. | Q8_4 |
|  | I made a movement. | Q8_5 |
| Competition | I was trying to win. | Q9_1 |
|  | I was trying to beat someone. | Q9_2 |
|  | I challenged myself. | Q9_3 |
|  | I was in competition. | Q9_4 |
|  | I felt challenged. | Q9_5 |
| Cooperation | I gave someone a hand. | Q10_1 |
|  | I cooperated with someone. | Q10_2 |
|  | I made decisions with someone. | Q10_3 |
|  | I acted together with someone. | Q10_4 |
|  | I achieved a goal with someone. | Q10_5 |
| Prosociality | I took care of someone/something. | Q11_1 |
|  | I helped someone. | Q11_2 |
|  | I did good for others. | Q11_3 |
|  | I put others’ interests before mine. | Q11_4 |
|  | I took care of someone. | Q11_5 |
| Aggression | I felt aggressive. | Q12_1 |
|  | I felt violent. | Q12_2 |
|  | I felt like dominating others. | Q12_3 |
|  | I felt like subduing others. | Q12_4 |
|  | I felt like destroying others. | Q12_5 |
| Spontaneity | I was spontaneous. | Q13_1 |
|  | I was authentic. | Q13_2 |
|  | I was myself. | Q13_3 |
|  | I had not planned it. | Q13_4 |
|  | I had not foreseen it. | Q13_5 |

Table S1. Final set of 65 play perception items derived from item generation process.

| **Item description** | **Item** | **Standardized Factor Loadings** | | | | | | |
| --- | --- | --- | --- | --- | --- | --- | --- | --- |
|  |  | **F1** | **F2** | **F3** | **F4** | **F5** | **F6** | **F7** |
| I had a good time. | Q1_2 | .680 |  |  |  |  |  |  |
| I enjoyed the moment. | Q1_4 | .635 |  |  |  |  |  |  |
| I liked it. | Q1_3 | .609 |  |  |  |  |  |  |
| I was focused. | Q2_3 | .354 |  | .253 |  | .298 |  |  |
| I was alert. | Q2_4 | .356 |  |  |  | .219 |  |  |
| I was attentive. | Q2_2 | .362 |  |  |  | .229 |  |  |
| I was enthusiastic. | Q3_3 | .591 |  |  |  |  |  |  |
| I was excited. | Q3_2 | .484 | .239 |  |  |  |  |  |
| I was stimulated. | Q3_1 | .428 |  |  |  |  |  |  |
| I learned something. | Q4_1 |  |  |  |  | .736 |  |  |
| I acquired knowledge. | Q4_4 |  |  |  |  | .741 |  |  |
| I understood something. | Q4_5 |  |  |  |  | .412 |  |  |
| I could choose what to do. | Q5_4 |  |  |  |  |  |  | .669 |
| I could do whatever I wanted. | Q5_5 |  |  |  |  |  |  | .603 |
| I was free. | Q5_1 | .299 |  |  |  |  |  | .336 |
| I created something. | Q6_1 |  | .771 |  |  |  |  |  |
| I built something. | Q6_2 |  | .702 |  |  |  |  |  |
| I used artistic skills. | Q6_5 |  | .631 |  |  |  |  |  |
| I used my imagination. | Q7_1 |  | .470 |  |  |  |  |  |
| I invented something. | Q7_5 |  | .746 |  |  |  |  |  |
| I had an open mind. | Q7_2 | .341 |  |  |  |  |  |  |
| I made a movement. | Q8_5 |  |  |  |  |  | .793 |  |
| I moved. | Q8_1 |  |  |  |  |  | .805 |  |
| I changed position. | Q8_2 |  |  |  |  |  | .588 |  |
| I was in competition. | Q9_4 |  | -.215 | .635 |  |  |  |  |
| I felt challenged. | Q9_5 |  |  | .699 |  | .201 |  |  |
| I was trying to win. | Q9_1 |  | -.332 | .561 |  |  |  |  |
| I cooperated with someone. | Q10_2 |  |  |  | .747 |  |  |  |
| I acted together with someone. | Q10_4 |  |  |  | .778 |  |  |  |
| I made decisions with someone. | Q10_3 |  |  |  | .779 |  |  |  |
| I helped someone. | Q11_2 |  |  |  | .595 |  |  |  |
| I did good for others. | Q11_3 |  |  |  | .432 |  |  |  |
| I took care of someone/something. | Q11_1 |  | .471 |  | .284 |  |  |  |
| I felt like subduing others. | Q12_4 |  |  | .802 |  |  |  |  |
| I felt aggressive. | Q12_1 |  |  | .718 |  |  |  |  |
| I felt like destroying others. | Q12_5 |  |  | .821 |  |  |  |  |
| I was authentic. | Q13_2 | .263 | .233 |  |  | .226 |  | .232 |
| I was spontaneous. | Q13_1 | .353 |  |  |  |  |  | .380 |
| I had not planned it. | Q13_4 |  |  |  |  |  |  | .284 |

Table S2. Standardized factor loadings of the 39 initial items across the seven-factor solution prior to item selection, using minres extraction and oblimin rotation (exploratory factor analysis). *Note.* F = Factor.

| **Item description** | **Item** | **Standardized Factor Loadings** | | | | | | | |
| --- | --- | --- | --- | --- | --- | --- | --- | --- | --- |
|  |  | **F2** | **F1** | **F4** | **F7** | **F3** | **F6** | **F8** | **F5** |
| I had fun | Q1_1 |  | .701 |  |  |  |  |  |  |
| I had a good time | Q1_2 |  | .630 |  |  |  |  |  |  |
| I liked it | Q1_3 |  | .692 |  |  |  |  |  |  |
| I enjoyed the moment | Q1_4 |  | .574 |  |  |  |  |  |  |
| I relaxed | Q1_5 |  | .264 |  | .325 |  |  |  |  |
| I was concentrated | Q2_1 |  | .389 |  |  |  |  |  |  |
| I was attentive | Q2_2 |  | .299 |  |  |  |  |  |  |
| I was focused | Q2_3 |  | .292 |  | .244 |  |  |  | .325 |
| I was alert | Q2_4 |  |  |  | .423 |  |  |  |  |
| I was clear-headed | Q2_5 |  |  |  | .460 |  |  |  |  |
| I was stimulated | Q3_1 |  | .349 |  | .213 |  |  |  | .280 |
| I was excited | Q3_2 |  | .386 |  |  |  |  |  |  |
| I was enthusiastic | Q3_3 |  | .527 |  |  |  |  |  |  |
| I was thrilled | Q3_4 |  | .248 |  |  | .220 |  |  |  |
| I was energetic | Q3_5 |  | .369 |  |  |  | .273 |  |  |
| I learned something | Q4_1 | .229 |  |  |  | -.239 |  |  | .504 |
| I thought about something | Q4_2 |  |  |  | .260 |  |  |  |  |
| I reflected on something | Q4_3 | .292 |  |  | .365 |  |  | .251 |  |
| I acquired knowledge | Q4_4 |  |  |  |  |  |  |  | .603 |
| I understood something | Q4_5 |  |  |  |  |  | .227 |  | .337 |
| I was free | Q5_1 |  | .316 |  | .302 |  |  |  |  |
| I had no constraints | Q5_2 | .235 |  |  | .284 |  |  | -.272 |  |
| I was autonomous | Q5_3 |  | .283 |  | .269 |  |  |  |  |
| I could choose what to do | Q5_4 |  |  |  | .565 |  |  |  |  |
| I could do whatever I wanted | Q5_5 |  |  | -.225 | .313 | .324 |  | -.309 |  |
| I created something | Q6_1 | .751 |  |  |  |  |  |  |  |
| I built something | Q6_2 | .665 |  |  |  |  |  |  |  |
| I used my imagination | Q6_3 | .664 |  |  |  |  |  |  |  |
| I experimented | Q6_4 | .436 |  |  |  |  |  |  | .317 |
| I used artistic skills | Q6_5 | .572 |  |  |  |  |  | -.205 |  |
| I used my imagination | Q7_1 | .588 |  |  |  |  |  |  |  |
| I had an open mind | Q7_2 | .303 | .314 |  |  |  |  |  |  |
| I thought freely | Q7_3 |  | .353 |  | .441 |  |  |  |  |
| I thought outside the box | Q7_4 | .247 |  |  |  | .202 |  |  |  |
| I invented something | Q7_5 | .800 |  |  |  |  |  |  |  |
| I moved | Q8_1 |  |  |  |  |  | .790 |  |  |
| I changed position | Q8_2 |  |  |  |  |  | .664 |  |  |
| I moved my hands | Q8_3 |  |  |  | .207 |  | .300 |  |  |
| I moved around | Q8_4 |  |  |  |  |  | .735 |  |  |
| I made a movement | Q8_5 |  |  |  |  |  | .816 |  |  |
| I was trying to win | Q9_1 | -.271 |  |  |  |  |  | .634 |  |
| I was trying to beat someone | Q9_2 |  |  |  |  |  |  | .606 |  |
| I challenged myself | Q9_3 |  |  | -.204 |  |  |  | .342 | .223 |
| I was in competition | Q9_4 |  |  |  |  |  |  | .711 |  |
| I felt challenged | Q9_5 |  |  |  |  | .285 |  | .603 |  |
| I gave someone a hand | Q10_1 |  |  | .715 |  |  |  |  |  |
| I cooperated with someone | Q10_2 |  |  | .712 |  |  |  |  |  |
| I made decisions with someone | Q10_3 |  |  | .729 |  |  |  |  |  |
| I acted together with someone | Q10_4 |  |  | .687 |  |  |  |  |  |
| I achieved a goal with someone | Q10_5 |  |  | .559 |  | .215 |  |  | .226 |
| I took care of someone/something | Q11_1 | .402 |  | .363 |  |  |  |  |  |
| I helped someone | Q11_2 |  |  | .676 |  |  |  |  | -.203 |
| I did good for others | Q11_3 |  |  | .529 |  |  |  |  |  |
| I put others’ interests before mine | Q11_4 |  |  | .417 |  |  |  |  |  |
| I took care of someone | Q11_5 |  |  | .356 |  |  |  |  |  |
| I felt aggressive | Q12_1 |  |  |  |  | .850 |  |  |  |
| I felt violent | Q12_2 |  |  |  |  | .781 |  |  |  |
| I felt like dominating others | Q12_3 |  |  |  |  | .564 |  | .339 |  |
| I felt like subduing others | Q12_4 |  |  |  |  | .695 |  | .257 |  |
| I felt like destroying others | Q12_5 |  |  |  |  | .654 |  | .281 |  |
| I was spontaneous | Q13_1 |  | .266 |  | .403 |  |  |  |  |
| I was authentic | Q13_2 | .242 |  |  | .395 |  |  |  |  |
| I was myself | Q13_3 |  | .353 |  |  |  |  |  |  |
| I had not planned it | Q13_4 |  |  |  | .525 |  |  |  | -.253 |
| I had not foreseen it | Q13_5 |  |  |  | .462 |  |  |  | -.315 |

**Table S3**. Replication of EFA on the full set of 65 items.
*Note.* F = Factor.

| **# Item** | **Item description** | **SD** | **IFC** | **KMO** | **Cross-loadings** |
| --- | --- | --- | --- | --- | --- |
| 1 | I enjoyed the moment. | .870 | .462 | .870 | 28, 41, 46 |
| 2 | I was enthusiastic. | .854 | .518 | .860 | 19, 28, 41 |
| 3 | I was excited. | 1.155 | .394 | .810 |  |
| 4 | I was stimulated. | 1.123 | .428 | .900 | 19, 21, 28, 41, 43 |
| 5 | I created something. | 1.459 | .538 | .870 |  |
| 6 | I used artistic skills. | 1.377 | .499 | .890 |  |
| 7 | I used my imagination. | 1.370 | .509 | .850 | 39 |
| 8 | I invented something. | 1.422 | .597 | .830 |  |
| 9 | I took care of someone/something. | 1.3487 | .373 | .830 | 18 |
| 10 | I was in competition. | 1.430 | .461 | .780 |  |
| 11 | I felt challenged. | 1.422 | .461 | .820 |  |
| 12 | I felt the urge to dominate others. | 1.265 | .418 | .780 | 29, 31 |
| 13 | I felt the urge to destroy others. | 1.306 | .489 | .800 |  |
| 14 | I cooperated with someone. | 1.399 | .389 | .760 |  |
| 15 | I acted together with someone. | 1.320 | .445 | .750 |  |
| 16 | I made decisions with someone. | 1.314 | .387 | .780 |  |
| 17 | I helped someone. | 1.231 | .471 | .750 |  |
| 18 | I did something good for the other. | 1.158 | .293 | .760 | 9 |
| 19 | I learned something. | 1.161 | .637 | .820 | 2, 4 |
| 20 | I acquired knowledge. | 1.154 | .644 | .830 |  |
| 21 | I understood something. | 1.116 | .622 | .870 | 4 |
| 22 | I made a movement. | 1.326 | .508 | .570 |  |
| 23 | I moved. | 1.322 | .541 | .600 |  |
| 24 | I changed position. | 1.332 | .398 | .800 |  |
| 25 | I could choose what to do. | 1.053 | .459 | .850 | 44 |
| 26 | I could do what I wanted. | 1.256 | .459 | .840 | 45 |
| 27 | I was free. | .950 | .452 | .900 | 45 |
| 28 | I was spontaneous. | .936 | .310 | .910 | 1, 2, 4, 43, 46 |
| 29 | I started arguing and fighting. | 1.213 | .431 | .870 | 12, 35 |
| 30 | I did not respect turns. | 1.098 | .363 | .810 |  |
| 31 | I disagreed with others. | 1.132 | .395 | .890 | 12 |
| 32 | I rejected others’ play ideas. | 1.073 | .368 | .820 |  |
| 33 | I cheated. | 1.128 | .233 | .700 |  |
| 34 | I felt confused. | .942 | .318 | .800 |  |
| 35 | I was ignored by others. | 1.104 | .405 | .820 | 29 |
| 36 | I isolated myself from the person/group I was playing with. | .972 | .318 | .830 |  |
| 37 | I refused to play when asked. | 1.139 | .394 | .820 |  |
| 38 | I withdrew from the game. | 1.161 | .387 | .850 |  |
| 39 | I was completely absorbed in my thoughts. | 1.343 | .277 | .840 | 7 |
| 40 | I lost track of time. | 1.269 | .305 | .810 |  |
| 41 | I was totally focused on what I was doing. | .990 | .359 | .830 | 1, 2, 4 |
| 42 | My thoughts were flowing smoothly. | 1.031 | .232 | .850 |  |
| 43 | I was totally immersed in what I was doing. | 1.157 | .429 | .830 | 4, 28 |
| 44 | I felt in full control of what I was doing. | .966 | .288 | .840 | 25 |
| 45 | I was playing without limitations. | 1.322 | .309 | .790 | 26, 27 |
| 46 | I knew what to do in the game. | .787 | .216 | .840 | 1, 28 |

Table S4. Summary of item-level psychometric properties prior to CFA.

*Note.* SD *=* standard deviation; IFC = intra-factor consistency; KMO = Kaiser-Meyer-Olkin value.
